# Dorsal and ventral stream visuoperceptual processing deficits in Lewy body disease

**DOI:** 10.1007/s00415-026-14166-5

**Published:** 2026-09-25

**Authors:** Emily McCann, Felicia Coleman, Soohyun Lee, Amir Fazlollahi, John D. O’Sullivan, Peter J. Nestor

**Affiliations:** 1 Clem Jones Centre for Ageing Dementia Research, Queensland Brain Institute, The University of Queensland, St Lucia, QLD 4072 Australia; 2 School of Health and Rehabilitation Sciences, The University of Queensland, St Lucia, QLD 4072 Australia; 3 Cognitive Health Program, Mater Research Institute, The University of Queensland, South Brisbane, QLD 4101 Australia; 4 Centre for Clinical Research, Faculty of Health, Medicine, and Behavioural Sciences, The University of Queensland, Herston, QLD 4029 Australia; 5 Department of Neurology, Royal Brisbane & Women’s Hospital, Herston, QLD 4029 Australia; 6 Department of Neurology, Mater Adult Hospital, Mater Misericordiae Limited, South Brisbane, QLD 4101 Australia

**Keywords:** Dementia, Visual impairments, Neuropsychology, Cognition, Dorsal stream, Ventral stream

## Abstract

**Introduction:**

Hypometabolism of visual-processing cortices is the hallmark of dementia in Lewy body diseases (LBD) yet visuoperceptual impairments remain under-researched compared to other cognitive domains.

**Methods:**

Novel, sensitive measures of dorsal- and ventral-stream visual processing were developed and administered to *n*=53 LBD patients, spanning cognitively asymptomatic to dementia, and compared to *n*=29 Alzheimer’s disease (AD) patients. Further analyses stratified LBD for visual hallucination status and contrasted visuoperceptual performance to measures of attention/executive function**.**

**Results:**

Visuoperceptual impairments were more prominent in LBD than AD, being detectable even in some that were cognitively asymptomatic. LBD demonstrated comparable ventral and dorsal impairments, whereas ventral visuoperceptual performance was relatively preserved compared to dorsal in AD. In LBD, visuoperceptual impairments were strongly associated with visual hallucination and explained a significant amount of performance on visually presented executive function/attention measures. The effect sizes of impairment in demented LBD were substantially larger on the novel visuoperceptual tests compared to standard non-visually presented tests of attention (Digit Span) and executive function (phonemic fluency).

**Discussion:**

Early dorsal and ventral stream impairments are a hallmark of dementia in LBD, differing quantitatively and qualitatively from AD. These deficits in LBD were more pronounced than those of attention and executive function.

## Introduction

Lewy body disease (LBD)—subdivided into Parkinson’s disease (PD) and dementia with Lewy bodies (DLB)—gives rise to the second most common form of degenerative dementia [1]. Dementia is, obviously, the hallmark of DLB, while over 80% of people with PD will develop dementia (PDD), though this may take more than a decade to emerge [2]. The development of dementia in LBD is associated with hypometabolism, as measured by fluorodeoxyglucose positron emission tomography (PET) [3, 4], and reduced cholinergic PET ligand binding [3] in posterior isocortical regions; these regions process visual information. Visuoperceptual processing impairments are an acknowledged feature of LBD dementia—as is visual hallucination [4, 5]—but they are under-researched, particularly in comparison to attention and executive function [4–6].

There are problems with existing tests of visuoperceptual abilities for LBD assessment. Bedside tests such as clock drawing or copying pentagons are useful screening tests but lack dynamic range—having both ceiling and floor effects—to understand the trajectory of decline; they also require a motor response that may be confounded by the movement disorder of LBD. Pure visuoperceptual tasks used in neuropsychology, such as the Benton Judgment of Line Orientation (JLO) [7] or the Visual Object and Space Perception (VOSP) [8], resolve the motor confound but suffer from ceiling effects, meaning they may be insensitive to early deficits.

To characterize the visuoperceptual deficit in LBD, two new tests were developed to probe dorsal and ventral streams of visual processing [9]. These tests were designed, devoid of ceiling effects, to be sensitive to the earliest signs of impairment. Alzheimer’s disease (AD) is also associated with a posterior temporoparietal hypometabolic pattern [10, 11] and so would also be expected to be associated with impairments on these tasks. People with AD were, therefore, included to contrast their visual processing performance to that of LBD in terms of the stage at which deficits might emerge, and to determine whether qualitative differences in ventral and dorsal stream involvement might exist between the two disorders.

## Materials and methods

### Participants

The study participants were *n* = 53 with LBD comprising cognitively asymptomatic PD (*n* = 27) through to varying severities of PDD or DLB (*n* = 26), and *n* = 29 with typical, memory onset, Alzheimer’s disease (AD). All patient diagnoses were confirmed by experienced neurologists (JDOS, PJN) in accordance with diagnostic criteria for AD [10], PD [12], PDD [5], and DLB [4]. Those classified as PD were cognitively asymptomatic and endorsed as such by their informant/caregiver. All patients with AD were biomarker confirmed with cerebrospinal fluid examination and spanned a range from the mild cognitive impairment disease stage through to moderately advanced dementia. For some analyses, patients with DLB and PDD were combined into a single group due to shared clinical symptoms and neuropathology [13]. All patients were tested while on their usual medication, and those with an LBD were always tested in the ‘on’ state. To account for any possible impact of cognitive fluctuations or delirium in the LBD group, the cognitive assessment only proceeded after confirmation from the informant/caregiver at the start of the test session that the patient was functioning at their usual mental state. LBD patients were also classified based on hallucination status. They were assigned to the hallucinator category when they reported a history of well-formed, complex visual hallucinations, corroborated by their caregivers. Patients were still classified in the hallucinator group if previously experienced visual hallucination had been suppressed by medication. A Snellen chart was used to measure all participants’ visual acuity. The International Standard Classification of Education (ISCED) [14] was used to categorize level of education. Patient demographics are presented in Table 1.

**Table 1:** Patient demographics

	Control	AD	PD	DLB-PDD	Omnibus significance
*n*	54	29	27	26	
Age	69.1 ± 5.0	67.6 ± 7.7	68.7 ± 7.7	73.5 ± 5.3	
Sex (male|female)	23 | 31	14 | 15	20 | 7	21 | 5	0.003^ab^
Education (ISCED)	5.4 ± 2.0	4.0 ± 1.9	4.6 ± 1.9	3.8 ± 2.3	< 0.001^bc^
Visual acuity (median [range])	6/6 [6/5—6/18]	6/6 [6/5—6/18]	6/9 [6/5—6/24]	6/9 [6/6—6/36]	0.128
National Adult Reading Test—NART (/50)		30.9 ± 7.6	33.1 ± 7.1	33.2 ± 7.9	0.25
Symptom duration		3.3 ± 2.0	5.3 ± 5.6	6.1 ± 4.7	0.047^b^
History of visual hallucination (%)		0	15	62	
*Medications*					
Cholinesterase inhibitor—ChI (%)		52	22	65	
Levodopa Equivalent Daily Dose—LEDD			622.3±933.7	161.0±204.1	0.001^f^
*Hospital Anxiety and Depression Scale—HADS*					
Depression (/21)		4.4 ± 3.4	4.3 ± 2.4	5.8 ± 3.0	0.116
Anxiety (/21)		5.3 ± 3.2	6.1 ± 3.1	6.5 ± 3.4	0.37^§^
*Motor symptoms*					
Hoehn and Yahr (/5)			1.5 ± 0.4	1.7 ± 0.4	0.033^g^

^§^ANOVA

^a^Control < DLB-PDD

^b^AD < DLB-PDD

^c^Control versus PD

^d^Control versus DLB-PDD

^e^Control > AD

^f^PD > DLB-PDD

^g^PD < DLB-PDD

In keeping with the known greater prevalence of LBD in males [15], the proportion of males in the PD and DLB-PDD groups was higher than in the control and AD groups. The healthy control group was significantly more educated compared to AD and DLB-PDD. DLB-PDD had a longer symptom duration compared to AD. The PD group had a higher Levodopa Equivalent Daily Dose (LEDD), and lower Hoehn and Yahr (H&Y) scores compared to DLB-PDD. Consent was obtained according to the Declaration of Helsinki and was approved by the Mater Misericordiae Ltd Human Research Ethics Committee (EC00332). All participants gave written informed consent prior to participation.

### General neuropsychology

All participants completed the Addenbrooke’s Cognitive Examination III (ACE-III) [16] and both novel tests. Subgroups of patients also completed established neuropsychological tests of visuoperception, including the Judgment of Line Orientation (JLO) [7], and the Cube Analysis, Incomplete Letters, and Progressive Silhouettes subtests from the VOSP [8]. A further subgroup of *n* = 42 LBD patients also completed traditional neuropsychological tests of attention and executive function, namely Verbal Fluency [17, 18], the Trail Making Tests (TMT) [19], Forward and Backward Digit Span Test (Digit Span) [20] and the Digit Symbol Substitution Test (DSST) [20] in order to assess how visuoperceptual ability may impact test performance in these domains—specifically to examine whether tests of attention and executive function, when administered in the visual modality, might be confounded by visuoperceptual processing deficits.

### Novel neuropsychological tests of visuoperception

Novel neuropsychological tests of visuoperception were developed: the Graded Blurry Image (GBI) task to target the ventral visual stream, and the Simultanagnosia Test to target the dorsal visual stream. Both novel tasks were displayed on a 24-inch Dell Ultrasharp U2419H monitor, where stimuli subtended a visual angle of 1.89°. Participants were seated 90 cm from the screen while completing the tasks.

#### The Graded Blurry Image (GBI) test

**Fig. 1:**
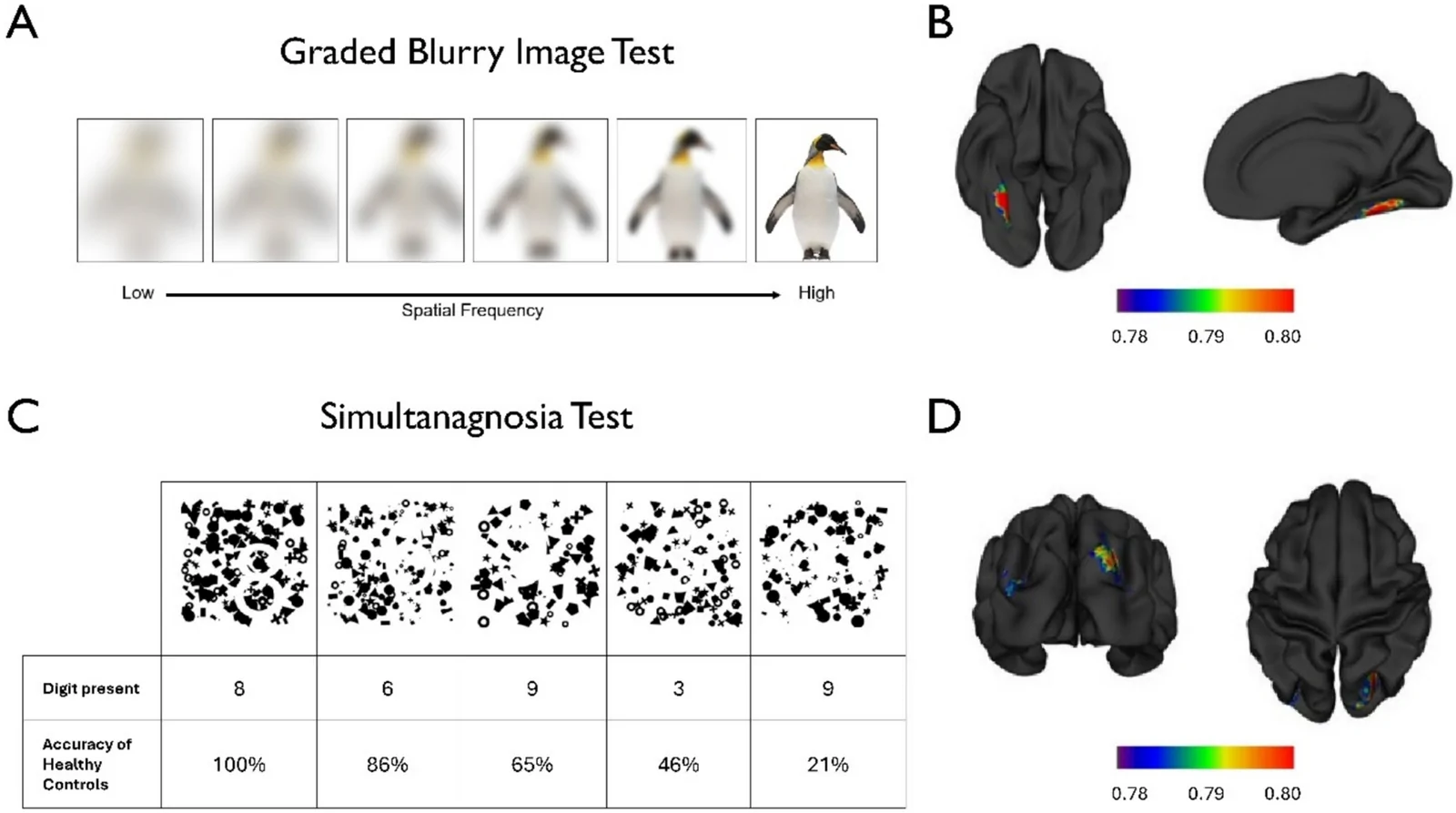
Examples of the novel tests and their construct validity. One item from the Graded Blurry Image test (**A**) and peak correlation of performance with cortical thinning in AD (**B**) and some exemplars from the Simultanagnosia Test (**C**) and its correlation with cortical thickness in AD (**D**)

The GBI is a 20-item test of object identification. Each item had inbuilt graded difficulty due to varying levels of image degradation. The items were blurred to varying degrees using Gaussian filters and presented in a coarse-to-fine order until the image was identified; larger Gaussian filters increased item difficulty. The initial draft version of the task comprised 44 items, each with 11 levels of blurring plus the unblurred image. Through a series of piloting iterations in healthy controls and patients, this was reduced to the final 20-item, plus two practice items, test by discarding items that subjects found too easy, too hard or where between-subject performance was highly variable. Each test item was also tuned to ensure participant time was not wasted in exposing them to excessive levels of impossible items: i.e., if, during piloting, no participant ever identified an object until the (e.g.) sixth hardest blur level, the hardest three items would be removed to save participants from being exposed to multiple redundant levels of difficulty. Difficulty was, therefore, specific to each item and determined by the earliest correct identification by healthy control participants in the pilot stage. Item-specific difficulty eliminated the possibility of ceiling and floor effects. To ensure aphasia was not confounding patient performance, accurate descriptions of the items were also accepted. More points were allocated for earlier identifications in the sequence: if there were six levels of degradation, a maximum of six points could be allocated. One point was allocated if the object was identified when undegraded, and zero for no identification. The final score was transformed into a percent-correct score per participant. Every item was identified by every patient who undertook this task. An exemplar from the task is shown in Fig. 1A.

#### The Simultanagnosia Test

The Simultanagnosia Test was created as a 40-item test of dorsal stream function. Simultanagnosia is the inability to process multiple visual elements at once.^28^ Creating the test initially involved the generation of hundreds of exemplars with varying amounts of random black noise in which a digit (from 0–9) was hidden (Fig. 1C) that the participant had to identify. The hidden digit was not a positive image; rather it was formed by the absence of background noise. Thus, varying the noise in each exemplar varied the amount of missing information of each digit, thereby creating a range of difficulties; simultanagnosia would make it more difficult to fill in this missing information to solve the task. The test was extensively piloted to create the final 40-item version in which healthy control performance on the exemplars ranged from 100% down to almost chance performance (Fig. 1C). Items were presented in progressing difficulty but with an easy item shown every eight items to maintain morale and task engagement. Participants had a maximum of 8 s to identify each hidden digit.

#### Construct validity and psychometric properties of novel tests

As cortical atrophy is typically not a prominent feature of dementia in LBD [21, 22], construct validity was determined by regressing scores on the novel tests with magnetic resonance imaging (MRI)-derived cortical thickness measurements in CSF biomarker-confirmed AD patients (n = 28 for GBI test; *n* = 31 for Simultanagnosia Test). To ensure a wide variance in test scores and cortical thickness values for robust regression analyses, the AD group included both typical and atypical (e.g., posterior cortical atrophy, corticobasal syndrome) clinical phenotypes.

MRI were acquired on a 3.0 T Siemens Prisma scanner (Siemens Healthineers, Erlangen, Germany) with a 64-channel head coil (software version VE11). Cortical atrophy was assessed using a 3-Dimensional (3D) Magnetization-Prepared Rapid Gradient-Echo Imaging (MPRAGE) sequence. The MPRAGE was acquired with 0.8mm^3^ spatial resolution; repetition/echo/inversion times = 2300/2.98/900 ms; flip angle 9°; field-of-view 256 × 192 × 256 mm; matrix size 256 × 192 × 256; bandwidth of 240 Hz/Px, IPAT2 with PE = 24; acquisition time 5:21 min.

The FreeSurfer 7.4.1 software was used to automatically segment cortical ribbon and generate surface meshes. The “recon-all” script was run with default parameters along with “-cm” and “-bg” flags to conform volumes to the acquired resolution (particularly essential for 0.8mm^3^ voxel size and addressing big ventricles when needed). The surface meshes were transformed to surface-based thickness maps using “-qcache -measure thickness -fwhm 15” flag. Test performance was then regressed against cortical thickness maps using Spearman’s *ρ*.

Peak statistical correlation of cortical thinning with performance on the GBI was in the ventral stream as expected: right ventral occipitotemporal region Spearman’s *ρ* = 0.83, *p* = 0.000001 (Fig. 1B). Peak statistical correlation with Simultanagnosia Test performance was in the dorsal stream: right posterior parieto-occipital junction Spearman’s *ρ* = 0.82, *p* = 0.00002 (Fig. 1D).

In healthy controls, performance on either the Simultanagnosia or GBI tests was not influenced by sex or education (*p* = 0.41 – 0.58). There was, however, a significant negative interaction with age: Simultanagnosia (*t* = − 2.4, *p* = 0.02) and GBI (*t* = − 3.1, *p* = 0.004).

### Statistical analysis

Statistical analyses were performed using RStudio version 4.2.0. Shapiro–Wilk tests were performed to determine data normality. If normally distributed, group-wise comparisons were performed using parametric one-way analysis of variance (ANOVA) with Tukey post hoc tests; if the data was not normally distributed, group-wise comparisons were performed using non-parametric Kruskal–Wallis tests with Dunn post hoc tests (omnibus statistics in the Tables are Kruskal–Wallis tests except where specified).

Regressions were run to determine how dementia severity, as measured by the global scale, ACE-III, related to performance on the novel tests, and if there were differential behaviors in patients with LBD and AD. In LBD, visuoperceptual performance was compared between those with, and those without, a history of visual hallucination, with the prediction that hallucinators would be significantly worse. To explore the possible impact of visuoperceptual impairment on traditional tests of executive and attentional abilities in LBD (Trail Making Test, Digit Symbol Substitution Test), multiple regressions were run to define the extent to which performance on these tests could be explained by visuoperceptual ability using the novel tasks versus tests of executive and attention that do not require visual input (Verbal Fluency, Digit Span). Performance on the novel visuoperception tests was compared to performance on traditional visuoperception tests.

## Results

### Performance on novel tests

The performance of control and patient groups on the GBI and Simultanagnosia Test is shown in Table 2.

**Table 2:** Performance (mean ± standard deviation) on novel tests across groups

Novel tests	Controls	AD	PD	DLB-PDD	Omnibus sig
Graded Blurry Images—GBI (%)	74.4 ± 4.9	66.1 ± 12.6	70.5 ± 9.3	60.3 ± 9.6	< 0.001^abcd^
Simultanagnosia Test (/40)	27.8 ± 4.8	14.9 ± 10.4	20.1 ± 9.1	10.2 ± 8.5	< 0.001^abde^

^a^Control > AD

^b^Control > DLB-PDD

^c^AD > DLB-PDD

^d^PD > DLB-PDD

^e^Control > PD

On the novel GBI, DLB-PDD patients had worse performance compared to healthy controls (*p* = < 0.001), AD (*p* = 0.035), and PD patient groups (*p* = 0.007). On the Simultanagnosia Test, AD (*p* < 0.001), PD (*p* = 0.014), and DLB-PDD (*p* < 0.001) had worse performance compared to healthy controls, but only DLB-PDD had worse performance compared to PD (*p* = 0.008).

Although at a group level on the GBI, there was no significant difference between the cognitively asymptomatic PD subgroup of LBD and controls, 30% of these patients registered a score below the − 1.5SD cutoff on this test. In the PD group, 44% of patients were below the − 1.5SD cutoff on the Simultanagnosia Test. PD patients who registered impairments on either test were significantly older than the PD patients who performed within the normal range (65.0 ± 8.7 years versus 72.1 ± 4.8 years, *p* = 0.01).

### Relationship to dementia severity

**Fig. 2:**
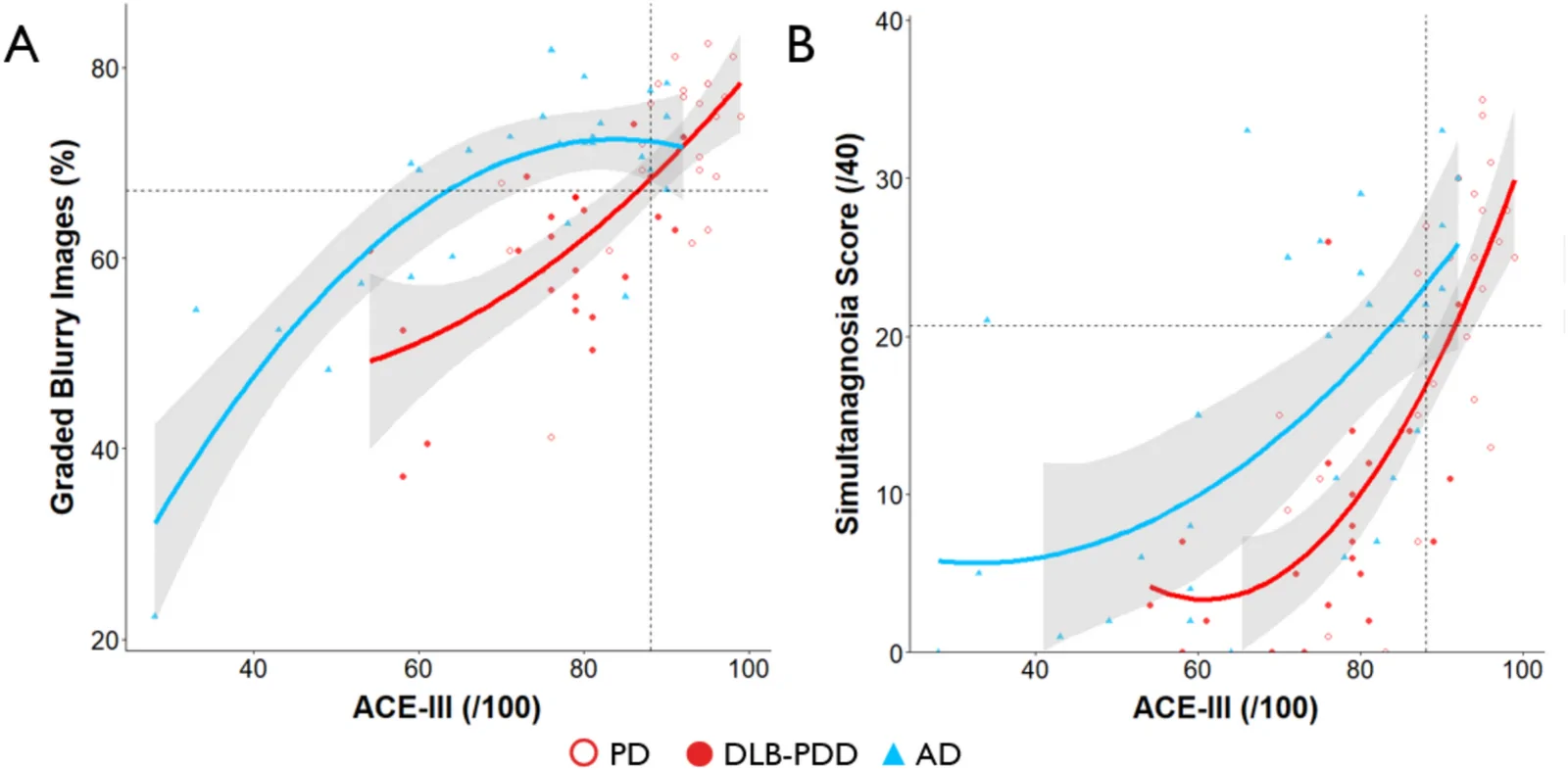
Quadratic models of test performance compared to ACE-III-defined dementia severity. *Note*. Dotted horizontal and vertical lines represent the cutoff score (worse than 1.5 standard deviations from the control mean) for impairment

To contextualize the timing of deficits on the novel tests in terms of dementia severity, quadratic regressions were run to compare performance in the LBD and AD groups against scores on the ACE-III (Fig. 2).

The analyses revealed both quantitative and qualitative differences between LBD and AD in the regression lines of GBI and ACE-III (Fig. 2A). Impaired performance on the GBI occurred at a much milder disease stage, as defined by the ACE-III, in LBD compared to AD. The regression line for LBD reached impaired GBI performance when scoring < 86.5/100 on the ACE-III; furthermore, there was a virtually linear relationship between the two measures. In contrast, performance on the GBI in AD was preserved through mild stages of dementia as defined by the global measure, with the regression line inflecting into the abnormal range on the GBI at an ACE-III score of < 63.4/100.

Impaired performance on the Simultanagnosia Test occurred at a milder ACE-III-defined disease stage in LBD compared to AD, but the regression lines were qualitatively similar for the two disease groups (Fig. 2B). The regression line in LBD crossed into the impaired range on the Simultanagnosia Test at an ACE-III score < 91.6/100; the regression line in AD reached impairment on the Simultanagnosia Test at < 83.8/100 on the ACE-III.

### Comparison to hallucination status

**Fig. 3:**
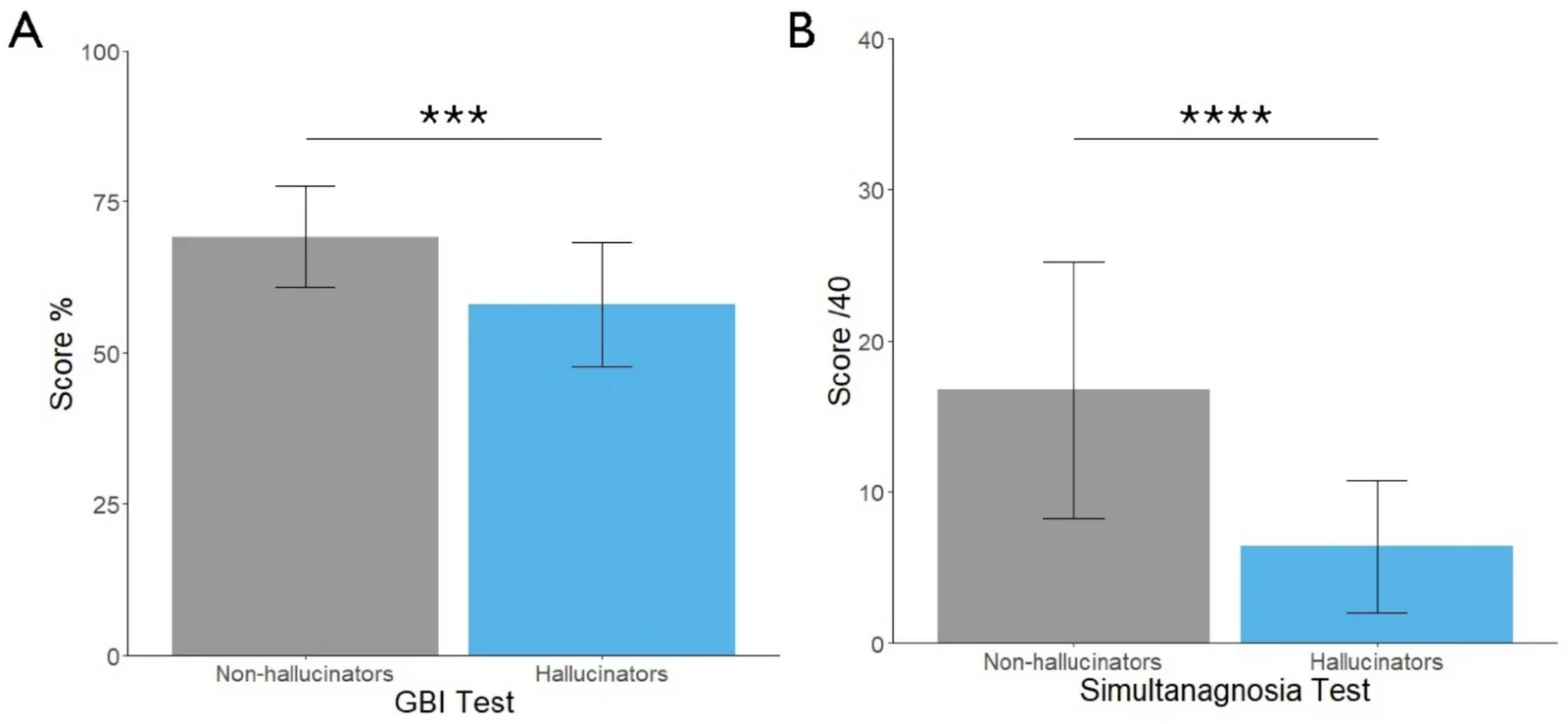
Comparison of performance on novel visual measures in LBD patients stratified for hallucination status. *Note.* **** *p* = 0.00003 *** *p* = 0.0003

LBD patients with a history of hallucination (*n* = 20) performed significantly worse than those without (*n* = 33) on the GBI, *t* = 3.99, *p* = 0.0003 (Fig. 3A). 88% of those with a history of hallucination scored in the impaired (worse than − 1.5SD) range on the GBI, while normal performance on the GBI had a negative predictive value for hallucination of 91%. For the Simultanagnosia Test, LBD patients with a history of hallucination also had significantly worse performance compared to those without, *t* = 4.66, *p* < 0.00003 (Fig. 3B). 95% of those with a history of hallucination scored in the impaired range on the Simultanagnosia Test, with normal performance on this test having a negative predictive value for hallucination of 95%.

### Comparison with attention/executive tests in LBD

It was hypothesized that performance on the DSST and TMT—common attention/executive function measures that are presented in the visual modality—might be significantly predicted by performance on the visuoperceptual tests, rather than on other tests of attention (Digit Span) and executive (phonemic fluency) that do not require visual processing. A subgroup of *n* = 42 LBD patients completed the DSST and TMT along with Digit Span and Letter (phonemic) Fluency as non-visual measures of attention and executive function, respectively. Descriptive statistics for this subgroup are shown in Table 3, with the results of multiple regressions of TMT and DSST in Table 4.

**Table 3:** Healthy control and LBD performance (mean ± standard deviation) on traditional neuropsychological tests of executive function/attention and the novel visual tests

	Controls	LBD	Omnibus statistic and significance
Novel visual tests			
GBI (%)	74.4 ± 4.9	67.6 ± 8.5	§F(1, 88) = 13.51, *p* < 0.001
Simultanagnosia Test (/40)	27.8 ± 4.8	17.1 ± 10.3	H(1) = 19.32, *p* < 0.001
Executive function			
Verbal Fluency			
* Letters*	15.0 ± 4.7	12.6 ± 5.8	§ F(1, 138) = 6.59, *p* = 0.011
Trail Making Test—TMT			
* Part A time (sec)*	28.7 ± 9.9	57.8 ± 47.8	H(1) = 17.38, *p* < 0.001
* Part A errors*	0.15 ± 0.5	0.41 ± 0.8	H(1) = 3.45, *p* = 0.06
* Part B time (secs)*	58.9 ± 18.2	144.7 ± 98.4	H(1) = 14.52, *p* < 0.001
* Part B errors*	0.59 ± 1.4	0.95 ± 1.6	H(1) = 0.87, *p* = 0.35
* Part B ÷ Part A (time)*	2.2 ± 0.8	2.6 ± 1.2	H(1) = 2.4, *p* = 0.12
* Part B – Part A (time)*	30.2 ± 16.9	86.9 ± 70.5	H(1) = 13.17, *p* < 0.001
Attention			
Digit Span (/28)	15.7 ± 3.5	14.2 ± 3.6	H(1) = 3.05, *p* = 0.081
Digit Symbol Substitution Test	52.7 ± 9.6	32.0 ± 14.9	H(1) = 39.96, *p* < 0.001

^§^ANOVA

**Table 4:** Results of the multiple regression analyses for Trail Making Test Part B and the Digit Symbol Substitution Test performance

Variable	β	SE	95% CI [LL, UL]	*t*	*p*
Trail Making Test Part B					
* Dorsal*					
Simultanagnosia Test	−0.81	1.15	[− 10.02, − 5.37]	−6.71	**< 0.001**
Digit Span	−0.01	3.37	[− 8.68, 4.96]	−0.55	0.584
Letter Fluency	0.05	1.98	[− 3.16, 4.87]	0.43	0.667
* Ventral*					
GBI	−0.69	1.24	[− 10.52, − 5.5]	−6.46	**< 0.001**
Digit Span	−0.19	3.27	[− 11.7, 1.54]	−1.55	0.129
Letter Fluency	−0.04	1.97	[− 4.7, 3.27]	−0.36	0.719
* Dorsal vs ventral*					
Simultanagnosia	−0.52	1.36	[− 7.7, − 2.22]	− 3.66	**< 0.001**
GBI	−0.37	1.64	[− 7.64, − 1.02]	−2.65	**0.011**
Trail Making Test (Time on Part B/Part A)					
* Dorsal*					
Simultanagnosia Test	−0.31	0.02	[− 0.08, 0.01]	−1.70	0.097
Digit Span	−0.24	0.06	[− 0.2, 0.04]	−1.32	0.196
Letter Fluency	−0.01	0.04	[− 0.08, 0.07]	−0.08	0.933
* Ventral*			[0, 0]		
GBI	−0.09	0.02	[− 0.06, 0.03]	−0.57	0.573
Digit Span	−0.34	0.06	[− 0.23, 0.01]	−1.89	0.066
Letter Fluency	−0.08	0.04	[− 0.09, 0.06]	−0.47	0.644
* Dorsal vs ventral*					
Simultanagnosia	−0.66	0.03	[− 0.13, − 0.02]	−2.82	**0.008**
GBI	0.25	0.03	[0.03, 0.1]	1.09	0.28
Trail Making Test (Time on Part B–Part A)					
* Dorsal*					
Simultanagnosia Test	−0.75	0.95	[− 7.06, − 3.21]	−5.4	**<0.001**
Digit Span	−0.07	2.79	[− 7.12, 4.2]	−0.52	0.604
Letter Fluency	0.10	1.64	[− 2.14, 4.52]	0.72	0.473
* Ventral*					
GBI	−0.58	1.09	[− 7.03, − 2.64]	−4.46	**<0.001**
Digit Span	−0.20	2.86	[− 9.79, 1.8]	−1.4	0.171
Letter Fluency	0.00	1.72	[− 3.49, 3.49]	0	1.000
* Dorsal vs ventral*					
Simultanagnosia	−0.56	1.20	[− 6.3, − 1.45]	−3.228	**0.003**
GBI	−0.22	1.45	[− 4.76, 1.09]	−1.267	0.213
Digit Symbol Substitution Test					
* Dorsal*					
Simultanagnosia Test	0.72	0.17	[0.69, 1.4]	5.97	**<0.001**
Digit Span	0.18	0.51	[− 0.3, 1.77]	1.43	0.161
Letter Fluency	−0.03	0.30	[− 0.69, 0.53]	−0.27	0.790
* Ventral*					
GBI	0.66	0.18	[0.8, 1.51]	6.55	**<0.001**
Digit Span	0.27	0.47	[0.17, 2.06]	2.39	**0.022**
Letter Fluency	0.04	0.28	[− 0.45, 0.68]	0.4	0.690
* Dorsal vs ventral*					
Simultanagnosia	0.48	0.21	[0.27, 1.11]	3.321	**0.002**
GBI	0.41	0.25	[0.21, 1.23]	2.863	**0.007**

Three different scoring systems for Trail Making Part B are presented: the raw score of Part B; Part B corrected score by dividing B by A; and Part B corrected by subtracting A from B

*Note*. Bolded *p* values indicate significance. The *β* weight is the standardized coefficient for the model

*CI* confidence interval, *GBI* Graded Blurry Image test, *LL* lower level, *UL* upper level

The TMT-B raw score was best explained by the dorsal stream task, with the Simultanagnosia Test explaining 64% of the variance (*F*(3, 38) = 25.65, *p* < 0.001). Re-running the regression with the Simultanagnosia Test substituted by the GBI yielded similar results, with the GBI explaining 63% of the variance in TMT-B performance (*F*(3, 38) = 24.35, *p* < 0.001). Correcting TMT-B by the method of subtracting TMT-A [23] produced broadly similar results, with performance on the Simultanagnosia Test explaining 52% of the variance (*F*(3, 38) = 15.96, *p* < 0.001). Similar results were again found for the GBI analyses, with performance significantly explaining 45% of the variance (*F*(3, 38) = 12.07, *p* < 0.001. Correcting TMT-B using the method of dividing by TMT-A [23] eliminated the contribution of the Simultanagnosia Test (*F*(3, 38) = 4.11, *p* = 0.01) and GBI (*F*(3, 38) = 2.97, *p* = 0.04).

For the DSST, the Simultanagnosia Test explained 63% of the variance in performance (*F*(3, 38) = 24.71, p < 0.001) with no significant contribution from Digit Span or Letter Fluency. The model using the GBI similarly explained 67% of the variance in DSST performance (*F*(3, 38) = 28.16, *p* < 0.001) with only a weak contribution from Digit Span and none from Letter Fluency. The Simultanagnosia Test and GBI each contributed comparably to performance on DSST.

The effect sizes in the DLB-PDD group compared to controls were larger for the novel visuoperceptual tests than for non-visually presented executive and attention tasks. The greatest effect size was seen on the Simultanagnosia Test (Cohen’s *d*: 1.88; corrected *p* < 0.0001) followed by the GBI (Cohen’s *d*: 1.55; corrected *p* = 0.0001) with lesser effect sizes for Letter Fluency (Cohen’s *d*: 0.72; corrected *p* = 0.001) and Digit Span (Cohen’s *d*: 0.57; corrected *p* = 0.062).

Likewise there was a large effect size on the Simultanagnosia Test between the PD group and controls (Cohen’s *d*: 0.62; corrected *p* = 0.003) with lesser effects for GBI (Cohen’s *d*: 0.38; corrected *p* = n.s.), Digit Span (Cohen’s *d*: 0.21; corrected *p* = n.s.) and no effect for Letter Fluency (Cohen’s *d*: 0.27; corrected *p* = n.s.).

### Comparison to dynamic range and effect sizes of existing visuoperceptual tests

No participant, including healthy controls, could perform at or near ceiling on the Simultanagnosia or GBI tests. Most participants also performed existing tests of visuoperceptual function, namely the selected subtests of the VOSP and JLO. Defining ceiling as ≥ 9/10 for the Cube Analysis subtest of the VOSP, *n* = 21/47 LBD and *n* = 4/17 AD patients were at ceiling; defining ceiling as ≥ 19/20 for the Incomplete Letters subtest of the VOSP, n = 18/47 LBD and 11/17 AD patients were at ceiling. The Progressive Silhouette subtest of the VOSP does not have a ceiling effect but was completely insensitive to detecting visuoperceptual deficits in the patient populations. Defining ceiling as ≥ 29/30 on the JLO test, *n* = 4/39 LBD, and *n* = 1/16 AD were at ceiling. The JLO also had the problem that n = 3/39 LBD and *n* = 1/16 AD failed the practice and, therefore, could not be administered the test.

*N*=4 LBD and *n*=2 AD patients recorded floor performance on the Simultanagnosia Test.

A subgroup of participants performed the novel and existing visuoperceptual at the same visit enabling a direct comparison of test performance. This analysis showed greater effect sizes for the novel tasks in identifying the visuoperceptual deficits in DLB-PDD compared to existing tests (Table 5).

**Table 5:** Performance (mean ± st dev.) of participants who performed both standard (VOSP subtests and JLO) and the novel visuoperceptual tests at the same visit, with effect sizes (Cohen’s d) of difference compared to controls

	Controls	AD	PD	DLB-PDD	Cohen’s *d* (versus controls)		
					AD	PD	DLB-PDD
Participants who performed both standard and novel dorsal stream tests	*	(*N*=14)	(*N*=16)	(*N*=24)			
JLO	25.20±3.78	20.5±8.7	24.06±4.85	17.33±7.62	0.71	0.27	1.32
VOSP Cube Analysis	9.33±0.93	7.5±2.18	9.41±0.71	6.88±2.61	1.10	0.09	1.26
Simultanagnosia	28.00±5.56	18.43±9.44	23.12±8.59	11.88±8.31	1.30	0.67	2.28
Participants who performed both standard and novel ventral stream tests	*	(*N*=15)	(*N*=19)	(*N*=24)			
VOSP Incomplete Letters	19.5±0.66	18.00±4.61	18.00±4.62	16.28±3.87	0.46	0.46	1.17
VOSP Silhouettes	10.50±3.22	10.20±3.67	10.00±2.4	10.88±2.82	0.08	0.17	0.13
Graded Blurry Images	74.3±4.40	71.52±8.95	70.52±10.91	61.85±8.96	0.39	0.45	1.76

*derived from in-house database of healthy volunteers aged 50–80 years (*N*=31 – 98 depending on the test)

## Discussion

The present study highlighted the prominence of visuoperceptual deficits in LBD, aligning with imaging research linking posterior isocortical hypometabolism to the emergence [24] and presence of dementia [3]. A key novel finding was that early visuoperceptual involvement in LBD affected both dorsal and ventral streams equally, whereas in AD, only dorsal impairments were evident in mild disease stages.

Impairments on simple visuoconstructive tasks, such as copying intersecting pentagons, have been shown to predict cognitive decline in PD [25], and were found to be a more prevalent early feature in DLB compared to AD [26]. While useful as screening tools, copying tasks lack dynamic range to understand the evolution of visuoperceptual deficits, and are potentially confounded by motor ability. Copying tasks also cannot determine whether specific aspects of visual processing may be preferentially affected or spared. Eliminating these confounds in the present study, DLB-PDD patients showed the greatest visual processing impairments on both novel measures despite having better global (ACE-III) scores than AD. This was consistent with previous imaging studies reporting greater posterior cortical hypometabolism in DLB-PDD than in AD [27, 28].

The relationship of visuoperceptual processing impairments to dementia stage revealed qualitative differences between AD and LBD. Whereas in LBD, the relationship between global cognition and both dorsal and ventral impairments was similar and emerged early, it was dissociated in AD with dorsal stream impairment being observed at very mild stage dementia (albeit not as mild as LBD), whereas ventral stream impairments occurred with moderately advanced dementia. Ventral stream findings were consistent with prior work using a degraded image approach reporting impairments in moderate, but not mild, AD [29], and with imaging studies that reported greater dorsal than ventral hypometabolism in AD [11, 30].

Significant impairment on the Simultanagnosia Test was observed in the cognitively asymptomatic PD group with 30% of this group also falling into the impaired range on the GBI. Performance on both tasks overlapped seamlessly between PD and DLB-PDD patients (but not with the AD group), consistent with the view that there is a continuum from normal cognition to dementia within LBD [1]. That a proportion of asymptomatic PD patients had impaired visuoperceptual processing aligns with past studies that identified posterior isocortical hypometabolism at predementia stages of LBD [31–33].

Visual hallucination is considered a predictor of emerging dementia in LBD [34]. Our results suggest that hallucination is not a *predictor,* but rather a *manifestation,* of the emerging dementia with the vast majority of LBD hallucinators scoring in the impaired range on both visuoperceptual tasks, while normal performance on either task had strong negative predictive value for hallucination history. Together, these findings indicate that visual processing impairments are, at least in part, likely driving hallucination in LBDs. Visual hallucination has been associated with posterior isocortical degeneration [35, 36] and visuoperceptual deficits [37–39]. Dissociated performance on the ventral stream task between LBD and AD groups suggests an interesting hypothesis regarding the emergence of hallucination. Visual hallucination in LBD mostly takes the form of people, animals, and objects—ventral stream functions [40, 41]. While visual hallucination is a core feature of dementia in LBD, it occurs late, or not at all, in AD—observations that resonate with the current ventral stream results. Furthermore, suppression of visual hallucination in LBD with cholinesterase inhibitors has been shown to focally improve cerebral perfusion in the ventral stream [36]. Dorsal stream dysfunction has also been linked to minor hallucinations of feeling someone being physically close (presence) or someone moving in the periphery (passage) [42].

Color perception is processed in the ventral stream [43], and thus the GBI results also resonate with reports of color perception deficits in LBD [44–46]—particularly research linking to visual hallucination [47]; impaired visuospatial abilities [47, 48]; and diffusion tensor MRI changes in white matter underlying visual association cortices [48]. In contrast, retinal pathology has been noted in LBD [49], and consequently, authors have highlighted that color perception [50, 51] and other visuoperceptual deficits [50, 52, 53] may be associated with this pathology. The possibility should be acknowledged that GBI impairments identified in LBD in this study may partly relate to retinal disease. The GBI was, however, specifically designed to minimise retinal pathology confounds by employing large, high-contrast images of objects that do not require detection of subtle variations in hue or contrast sensitivity. Moreover, we propose caution when attributing a causal role to retinal pathology for visuoperceptual deficits in LBD. For instance, the current results indicated that visuoperceptual deficits can be found in some PD patients who would not have been appreciated to have cognitive impairment or dementia, as defined by standard clinical measures. This, in turn, could potentially lead to the specious conclusion that visuoperceptual deficits in such individuals were due to retinal degeneration. Furthermore, evidence of hypometabolism and cholinergic deficits on PET in posterior cortical regions [3], and cholinesterase inhibitors ameliorating visual hallucination while focally improving cerebral perfusion in the ventral visual stream[36] are hard to reconcile with a retinal etiology. In contrast, such observations are fully compatible with posterior cortical visuoperceptual processing deficits.

The significance of the present results extends beyond understanding visuoperceptual function in LBD. Executive function and attention deficits are the most commonly reported in LBD [4–6], arguably, as they are the most studied [54]. As the motor syndrome of LBD is associated with degeneration of nigral dopaminergic projections to frontostriatal pathways, cognitive impairments have often been attributed to these frontal networks [55–57]. Measures used to expose executive-attention impairments in LBDs, however, frequently rely on visuoperceptual abilities. Similar to previous studies, we found that both PD and DLB-PDD demonstrated impairments on common measures of executive-attention abilities [58–61]. Performance on visually presented executive and attention tests (TMT-B and DSST) was, however, overwhelmingly explained by visuoperceptual impairments in LBD, with only the TMT-B/A correction method removing this factor.

Both TMT-B and the DSST depend on a wide range of abilities, including psychomotor speed, attention and working memory, associative learning, executive function, visual object recognition, and visuospatial location [23, 62]. Critically, however, considering the pre-eminence of the belief that frontostriatal dysfunction is a factor in understanding cognition in LBD, impaired performance on either test would likely have been interpreted historically as evidence of attention/executive dysfunction. The current results indicate that this interpretation would be incorrect. Furthermore, considering that 52% of the cognitively asymptomatic PD group in the current series scored in the impaired range on one or both novel visuoperceptual tasks, one could envisage a scenario in which attention and executive deficits in seemingly cognitively intact PD patients were spuriously attributed to frontostriatal networks by failing to accurately measure visuoperceptual ability. It was also notable that visual impairments identified by the novel tests were more severe (greater effect sizes) than non-visual executive-attention in LBD.

The main limitation of the study is that data was exclusively cross-sectional. Although visuoperceptual deficits were contextualized by comparison to ACE-III scores, a longitudinal study is required to establish the trajectories of decline. Lack of pathological confirmation in the LBD group is also a limitation. All participants in this group, however, were diagnosed by strict application of current clinical diagnostic criteria and were under longitudinal follow-up to ensure stability of diagnoses.

Visuoperceptual impairments, affecting both dorsal and ventral streams, are a hallmark early feature of LBD, detectable even in cognitively asymptomatic patients. These findings are strongly concordant with imaging literature linking the emergence of dementia in LBD to hypometabolism in posterior isocortical, vision-processing regions. More precise understanding of the evolution of cognitive impairments in LBD is crucial both for the diagnosis of emerging dementia and to reliably measure the impact of therapeutic interventions.

## Data Availability

The datasets generated and/or analyzed during the current study are not publicly available due to privacy reasons but are available from the corresponding author on reasonable request.
